# Health Literacy and Health Behavior Among Women in Ghazni, Afghanistan

**DOI:** 10.3389/fpubh.2021.629334

**Published:** 2021-03-04

**Authors:** Stefanie Harsch, Asadullah Jawid, Ebrahim Jawid, Luis Saboga-Nunes, Kristine Sørensen, Diana Sahrai, Uwe H. Bittlingmayer

**Affiliations:** ^1^Faculty of Educational Sciences, Institute of Sociology, University of Education Freiburg, Freiburg, Germany; ^2^American University of Afghanistan, Kabul, Afghanistan; ^3^Shuhada Hospital, Ghazni, Afghanistan; ^4^Faculdade de Medicina, Institute for Evidence Based Medicine, Universidade de Lisboa, Lisboa, Portugal; ^5^Coimbra Health School, PC, Coimbra, Portugal; ^6^Global Health Literacy Academy, Aarhus, Denmark; ^7^Institute of Special Pedagogy and Psychology, FHNW, University of Education, Muttenz, Switzerland

**Keywords:** Afghanistan, health-behavior, health literacy, HLS-EU-Q, illiterate, low-income country, women

## Abstract

**Background:** Health literacy is a determinant of health and assessed globally to inform the development of health interventions. However, little is known about health literacy in countries with one of the poorest health indicators worldwide, such as Afghanistan. Studies worldwide demonstrate that women play a key role in developing health literacy. Hence, this study's purpose is to explore health literacy of women in Afghanistan and the associated factors.

**Methods:** From May to June 2017, we randomly recruited 7–10 women per day at the hospital in Ghazni, a representative province of Afghanistan. Two trained female interviewers interviewed 322 women (15–61 years old) orally in Dari or Pashto on a voluntary basis and assessed their health literacy using the HLS-EU-Q16, associated socio-demographics, and health behavior.

**Results:** Health literacy of women (among educated and illiterates) is low even compared to other Asian countries. Health literacy is linked to age and education. We found mixed evidence of the relationship between health literacy and contextual factors, help-seeking, and health-related behavior.

**Conclusion:** This study provides novel data on health literacy and astonishing insights into its association with health behavior of women in Afghanistan, thus contributing to health status. The study calls for recognition of health literacy as a public health challenge be addressed in Afghanistan and other low-income countries affected by crises.

## Introduction

Recently, health literacy (HL) moved from the margins to the mainstream of health research because of its relevance for quality of care, disease prevention, and quality of life. Health literacy is a critical determinant of health, an asset for public health and an outcome of health promotion activities such as health education (1). Hence, researchers and policymakers recommend assessing health literacy, identifying vulnerable groups, and developing interventions to improve health literacy on this basis (2). Initially, most research originated from English-speaking countries (e.g., USA, Canada, and Australia), but other industrialized countries, such as many European Countries, quickly followed, and the countries of the global South and Asia are recently catching up (3–7). Nevertheless, crisis-affected and least-income countries are omitted, although assessing and improving health literacy there is supposed to contribute significantly to improving general health and the situation in the country and to achieving the sustainable development goals. Afghanistan is one of these most affected countries and at the same time places incredibly high challenges on research. Since—as far as we know—no health literacy research has been conducted in Afghanistan. Hence, we wanted to close the gap and explore the level of health literacy and provide relevant data for policymakers and practitioners and expand our knowledge of the nature of health literacy and related factors in war-torn and crisis-affected countries that have been neglected by health literacy research so far.

Various understandings of health literacy exist, the most prominent of which are those of the WHO (8), the Institute of Medicine (US) (9) and Sørensen et al., “Health literacy is closely linked to literacy and entails the knowledge, motivation and competences to access, understand, appraise, and apply information to form judgement and make decisions in terms of healthcare, disease prevention, and health promotion in everyday life during the life course.” (10) Health literacy is “more than the ability to read and comprehend health information” (11) and includes functional, interactive, and moreover critical health literacy (1). Consequently, numerous health literacy tools are available (12), such as performance-based screening tools for patients' functional health literacy, for example the Test of Functional Health Literacy of Adults (TOFHLA), the Rapid Estimate of Literacy in Medicine (REALM) and the Newest Vital Sign (NVS) (13–16) or self-reported health literacy tools that aim at capturing a more comprehensive understanding of health literacy, such as the European Health Literacy Questionnaire (HLS-EU-Q) and the Health Literacy Questionnaire (HLQ) (17, 18).

Besides differences in levels of health literacy between countries, research shows that a substantial proportion of people have difficulties dealing with health information even within each country worldwide (19). The European Health Literacy Survey indicated 30–63% of the population have limited health literacy, depending on the European country considered (7). This trend is reflected in the Asian Health Literacy Survey demonstrating similar or even worse results in six countries (20). The various studies on health literacy revealed the association of health literacy with certain determinants of health, primarily e.g., gender, age, education, financial deprivation, and social status (6, 21). Furthermore, studies identified health literacy as a determinant to health mediated by health-related choices and multiple health behaviors, including help-seeking behavior, reproductive health, and eating behavior (21–25).

However, little empirical evidence exists on the determinants of health, health behavior, or their interplay with health literacy in Afghanistan. Afghanistan's public health indicators are among the worst globally: e.g., infant mortality rate is 62/1,000, up to three-times higher than in neighboring countries (26). Due to the ongoing war since 1978, scientific studies are comparatively rare and even studies on health, reproductive health or healthy lifestyle are scarce in Afghanistan, almost non-existing in remote areas and, furthermore, data is doubtful. A high percentage of the population in Afghanistan is illiterate [on average, an estimated 38.2% of Afghans are literate [52% of men, 24.2% of women] (27)]. The group of Afghan women is unique because of the historical context and the drastic changes in women's access to education in recent decades. While girls were officially banned from attending schools in the 1990s, the attendance has increased considerably since 2001 (28, 29).

In health literacy research, a few studies target women in low and middle-income countries (30), but little is known about health literacy level in a population with interrupted or lacking education.

### Assumptions, Research questions, and Aims

Therefore, we wanted to examine the following assumptions: (a) Due to lack of/or interrupted school education, literacy and health literacy levels of many women in Afghanistan might be low. (b) Due to one of the lowest literacy rates worldwide, the level of health literacy in Afghanistan could be one of the lowest around the world. Hence, we used the HLS-EU-16 to facilitate comparability with findings from other countries, particularly in the European and Asian health literacy surveys (10, 20). (c) The health literacy level could be influenced by determinants namely higher educational attainment, younger age, higher socioeconomic status, living closer to the health center, and having better access to transportation and information and communication technologies (ICT). Access and ICTs can increase the likelihood of being exposed to more health information and thus contribute to improved health literacy (31, 32). (d) Bearing in mind the association of health literacy with education and age, age groups might differ in their health literacy level, a higher level of HL might be associated with seeking help at the hospital/medical center and with more health-promoting behavior. Therefore, our main research questions were:

- What is the health literacy level of women in Afghanistan?
- How high is Afghans' health literacy in comparison to other countries?
- What are the main determinants of health literacy in Afghanistan?
- Is health literacy associated with help-seeking and with health-related behavior?

Our aim is to present descriptive results of our health literacy survey in Afghanistan, to compare it with population health literacy of populations globally, and to discuss the complex relationship between literacy, health literacy, and health behavior for crisis-affected low-incoming countries. Notably, the study identifies practical approaches to meet the need to improve the population's health literacy and make healthcare, disease prevention, and health promotion more accessible to the people of Afghanistan (11, 33, 34).

## Data and Methods

### Research Area

We have conducted the survey in Hazarajat in four of the most densely populated districts of Ghazni province: Jaghori, Malistan, Nahoor, and Qarabagh. These districts are representative of the geography of Afghanistan with a study area of ~7,355 square kilometers and an altitude difference of more than 2,000 m. Only one hospital—the Shuhada Hospital—and 25 health centers operate in these districts.

### Sample and Data Collection

Various security-related, geographical, and cultural challenges in Afghanistan pose difficulties for research among Afghan women outside the main cities. Firstly, women should conduct interviews with women. Secondly, it was impossible for female interviewers to travel to villages and interview randomly selected women because of security reasons (35). Therefore, we have chosen the most suitable approach and interviewed women coming to Shuhada Hospital either for personal treatment or for accompanying a patient. For 2 months-−20 May−20 July 2017—we randomly selected 7–10 women per day aged 15 years or older. Due to the high number of illiterates, two trained female nurses and staff obtained oral informed consent and conducted the interviews orally on a voluntary basis. To ensure the highest attainable standard of participant safety, we took several measures. Interviews with women were conducted under close supervision of Shuhada Hospital management. Participation did not pose a safety risk because the subjects were interviewed in a designated office within the hospital. The interviewers explained that the data collected would be used for scientific research and would not include personal information such as name, address, and telephone number. They also informed respondents that they could refuse to answer any question if they did not feel comfortable doing so and that they could stop the interview at any time if they wished without any disadvantage. Apparently, respondents felt comfortable with the procedure and in the interview situation, as only a few respondents refused to answer some questions (mainly questions about satisfaction with sex life in the Quality of Life Questionnaire), but no woman ended the interview before completion.

Furthermore, we also interviewed male heads of households in their villages, but this sample is described in other articles (35, 36). The Ministry of Public Health in Afghanistan and the head of the Shuhada Hospital approved the study protocol.

### Questionnaire

Due to our desire to look at health and health literacy from various perspectives, we have created a questionnaire and selected questions based on the content, relevance and cultural appropriateness. The questionnaire comprised 102 items, including 45 socio-demographic and health-related items, 15 items of the European Health Literacy Questionnaire (HLS-EU-Q16), 18 items of Quality of Life (WHOQOL-BREF) (37), 8 items of the Spirituality, Religion, Personal Beliefs Questionnaire (WHOQOL-SRPB-BREF) (38), and 16 items of the Questionnaire for Patient Empowerment (39).

We used the HLS-EU-Q16 questionnaire developed by Pelikan et al. (40), shortened by Röthlin et al. (41) slightly modified and translated into Dari from the Swedish study by Wångdahl et al. (45) and translated this version into Pashto. To control the quality of our translation, we asked a heterogeneous group of people from Ghazni province if and how they understood the items and modified it based on their comments. The HLS-EU-Q16 focuses on how people find, understand, appraise, and apply information in three domains: health care, disease prevention, and health promotion. It consists of 16 items describing health-specific interactive tasks, the difficulty of which the interviewee is asked to rate on a four-point Likert scale: “very difficult” = 1, “rather difficult” = 2, “rather easy” = 3, or “very easy” = 4 with an additional option of “I don't know (18). In this study, we excluded one of the original 16 items: Item seven (“How easy/difficult is it for you to follow instructions from your doctor or pharmacist?”), which the Afghan field team considered irrelevant, since doctors have their own pharmacy in this district, so distinguishing between doctors and pharmacists could be confusing.

In general, the very high Cronbach's alpha α = 0.991 (items: 15, cases: 322) of the Health literacy scale can be regarded as valid for appropriate internal consistency of the items (7).

### Statistical Analysis

For data management, we adhered to the guidelines of the HLS-EU survey on inclusion and exclusion criteria and restricted the samples to participants 15 years and older, and to those answering at least 80% of the questions (41). Hence, we needed to exclude 2 out of 324 women because of their age (age 12 and 13), but no person was excluded due to missing values.

Besides descriptive analysis (range, minimum, maximum values of the items), we calculated the level of health literacy based on the mean value of the answers given per person. The score 1 symbolized the lowest mean score (= all items are very difficult) and 4 the highest mean score (= all items are very easy). Unlike the proposed procedure in the HLS-EU (40), we did not transform the 4-point Likert scale into a dichotomous scale. The reasons for this were that we would lose a great deal of variance within the responses by dichotomizing them. Other studies on people with low education show that in this group the loss of variance would be particularly strong and that we could not further investigate which determinants explain this existing difference. In addition, we assumed that the subjects had good reasons for selecting the respective level and thus we wanted to take their assessment into account. However, because our intention was to investigate the health literacy of women with no or little schooling, we considered the 4-point Likert scale to be the most appropriate. Next, we calculated the total mean HL score and its standard deviation and compared the total HL score with other countries. For this purpose, we standardized the HL score to a uniform metric of 0 to 50 as proposed by Röthlin et al. (41). The calculation formula is: Index = (mean-1)^*^50/3. Additionally, we subdivided the health literacy score of the short HLS-EU-Q into three levels at the cut-off of 25 and 37.4 points (equivalent to the subdivision of the HLS-EU-Q16 at 8 and 12 points). 0–25 points were defined to be the lowest level as “inadequate HL,” 25–37.4 as “problematic HL” and 37.5–50 as “sufficient HL” (7, 20, 40). We transformed the sociodemographic determinant “education” from a three categorical variable into a dichotomous variable (no education vs. some education) and occupation from a four categorical variable into a dichotomous variable (working at home vs. working outside, for instance, as teacher, governmental or NGO employee).

We examined the relationship between health literacy and other factors, namely age, education, further sociodemographic data, and health-related behaviors, by calculating bivariate correlation. Thereby we used pearson correlation coefficient *(r)* for two continuous-variables, point-biserial correlation coefficient *(rpb)* for one binary variable and one continuous-level variable, odds ratios *(OR)* for bimodal variables, and chi-square tests *(*χ^2^*)* for multi-optional variables using estimated percentage and absolute numbers. To assess the proportion of variance in HL, which is explained by sociodemographic data, we performed a multivariate linear regression model for general health literacy index as a dependent variable and education, age, main occupation (housewife or own occupation) and marital status as predictors.

## Results

A total of 322 women at the hospital from the following districts participated in the study: Jaghori (*N* = 242 women), Malistan (*N* = 77), Nahoor (*N* = 3), and Qarabagh (*N* = 2). At the time of the interview, 58.7% reported being sick, whereas 41.3% were not ill but were accompanying another person. The participants' age varied between 15 and 61 years (average 30.33), with more than half of the participants between 20 and 29 (*N* = 144) and only 26 women aged 50 and above.

Educational attainment was relatively poor. 59.6% women reported to be illiterate, 4.0% had basic education in reading and writing and approximately one out of three (36.3%) had formal or higher education.

Table 1 shows the characteristics of the sample. The majority of participants were married (83.2%) (Table 1), with 18-year-olds and younger people more likely to be single than married. 23.6% of women had no children, the majority reported up to nine pregnancies, and 16 women had 10–16 pregnancies. At the time of the interview, 20.2% were pregnant. Households were large with an average of 9.5 people (from 2 to 30) and an average of 3.1 literate persons. The most common occupation for 9 out of 10 women (89.1%) was “working at home,” a small number (3.1%) of the women were employed by the government or an NGO, and 1.2% were teachers. The main source of income cited by the women was remittances (25.2%), followed by farming (25.3%) and business (42.9%) and 6.5% salary when employed by the government or an NGO.

**Table 1:** Sample characteristics.

**Determinants**	**Female Patients/Attendees**
**Sample size**	*N* = 322
**Age** (years)Age groups	*M* = 30.33 (*R*: 15; 61) <20: *N* = 36 20 → 29: *N* = 144 30 → 39: *N* = 72 40 → 49: *N* = 44 50 ≤ : *N* = 26
**Marital status** (%)	82.7% married 17.3% single
**Education** (%)	59.6% illiterate 4.0% Basic reading & writing 36.3% formal education
**Profession** (main occupation) (%)	89.1% work at home 6.5% others 3.1% government/NGO employed 1.2% teachers
**Main source of income** (%)	42.6% business 25.6% remittances 25.3% farming 6.5% government/NGO employed
**Household size**	*M* = 9.56 (±5.3) (*R:* 2; 30)
**Number of literate people in household**	*M* = 3.18 (±2.13)
**Patients/Attendees** (%)	58.7% sick/patient 41.3% not sick: accompanying person

*N, absolute Numbers; M, Mean; R, Range; ±, Standard deviation*.

Concerning contextual factors, the women interviewed in the hospital reported that 41.0% had access to a river, 97.8% to a road and 43.8% to a car. Most, but not all women had access to information and communication technologies such as electricity (89.1%), phone (88.8%), TV (75.5%), and the Internet (23.3%).

### Health Literacy of Women in Afghanistan

**Figure 1:**
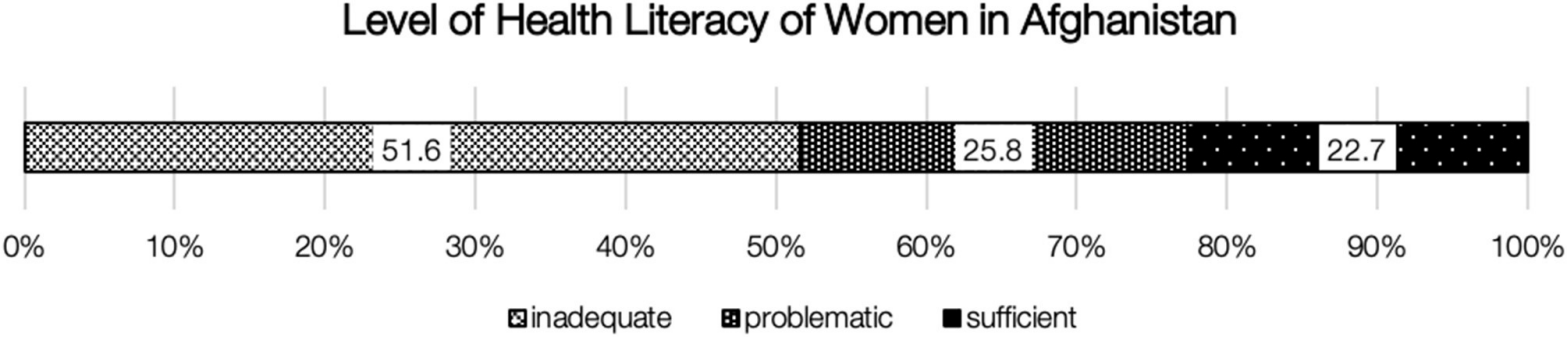
Level of health literacy of women in Afghanistan.

Figure 1 illustrates the women's level of health literacy. In our study, about half of all women (51.6%) had an inadequate level of health literacy as measured with the HLS-EU-Q16. One out of four women (25.8%) had problematic HL, and more than one-fifth of the women (22.7%) had sufficient HL.

Noteworthy differences exist in the self-reported responses related to the HLS-EU-Q16 scale, as some items (activities) were classified by the majority of respondents as easier and other items (activities) as more difficult. Women had the greatest difficulties with tasks related to evaluating recommendations e.g., “judging when to get a second opinion” (Item 5) (64.60% of women said it was rather or very difficult) and “judging which everyday behavior is related to health” (Item 16) (60.56%). However, most women (54.3%) reported that “understanding the advice on health given by family members” (Item 14) was the easiest of all tasks. Nevertheless, rarely all respondents found all items very/rather difficult or very/rather easy (see Table 2 on the single items and distribution).

**Table 2:** Overview of the European Health Literacy Survey Questionnaire 16 (HLS-EU-Q16) items and the distribution of the answers given by women in Afghanistan.

	**Items of the European Health Literacy Survey Questionnaire 16 (HLS-EU-Q16)** *“On a scale from one to four how easy do you find it to …?”*	**Mean (1–4)**	**SD**	**% (rather) easy**	**Confidence interval**
I1	Find information on treatments that concern you	2.33	1.16	47.35%	(41.86–52.84)
I2	Find out where to get professional help when ill	2.30	1.17	45.96%	(40.49–51.44)
I3	Understand what your doctor says	2.35	1.17	48.76%	(43.27–54.25)
I4	Understand instruction taking medication	2.48	1.2	55.59%	(50.13–61.05)
I5	Judge when to get second opinion	2.11	1.17	35.40%	(30.15–40.65)
I6	Use information doctor gives to make decisions	2.35	1.18	49.07%	(43.58–54.56)
I8	Find information on how to manage Mental health problems	2.18	1.13	40.68%	(35.29–46.08)
I9	Understand health warnings lifestyle	2.52	1.25	56.70%	(51.25–62.15)
I10	Understand why you need screening	2.34	1.15	49.69%	(44.20–55.18)
I11	Judge reliability of health information in media	2.44	1.19	54.35%	(48.88–59.82)
I12	Decide how to protect from illness based on media	2.44	1.2	53.42%	(47.94–58.89)
I13	Find out about activities good for mental well-being	2.23	1.14	40.68%	(35.29–46.08)
I14	Understand advice on health from family members or friends	2.41	1.17	54.35%	(48.88–59.82)
I15	Understand information in media on how to get healthier	2.46	1.2	54.66%	(49.19–60.12)
I16	Judge which everyday behavior is related to health	2.14	1.19	39.44%	(34.07–44.81)

### Health Literacy of Women in Afghanistan Compared to Other Countries

**Figure 2:**
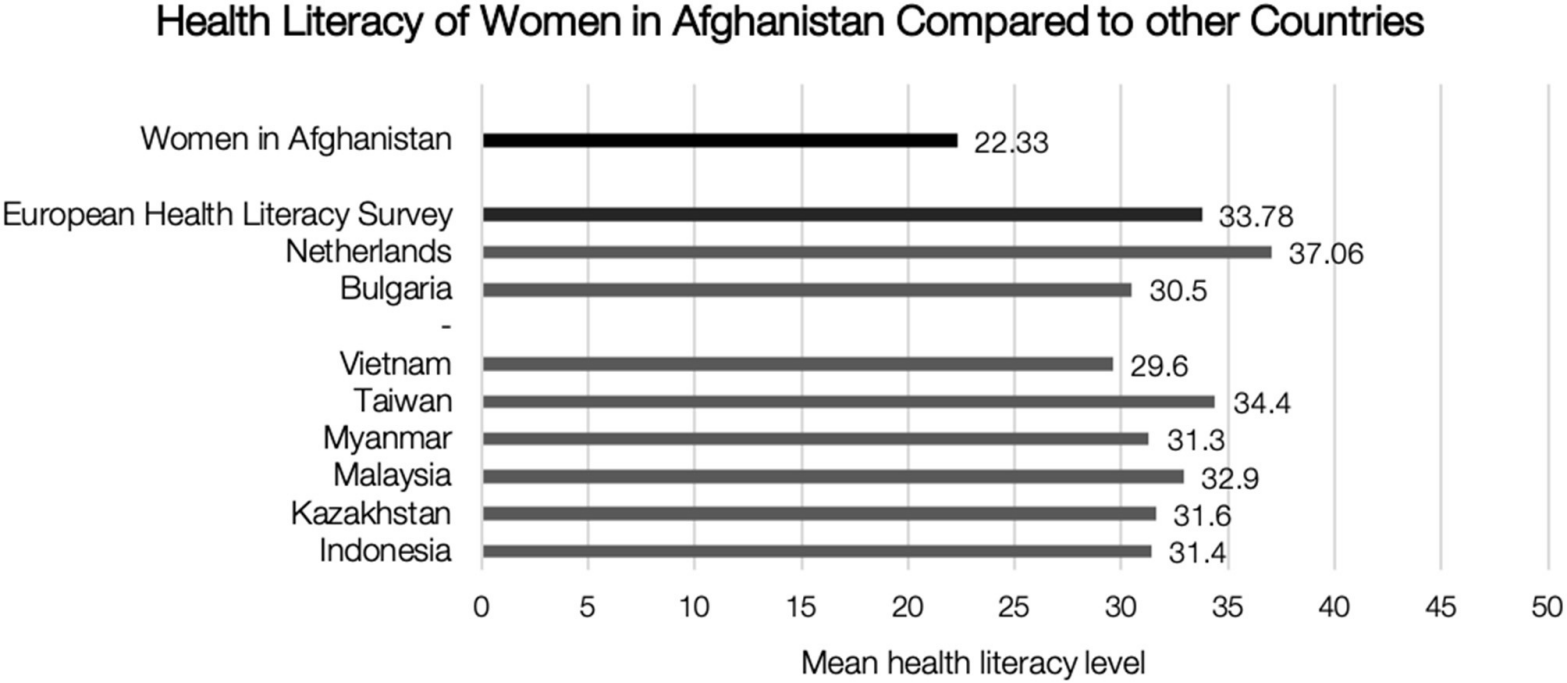
Health literacy of women in Afghanistan compared to other countries.

Figure 2 illustrates our data compared to the findings of two other studies. The first comparison refers to the six countries in the Asian survey using the HLS-EU-Q. The second is with three selected results from the original HLS-EU survey: the study's average, the country with the lowest HL level, Bulgaria, and the country with the highest HL level, the Netherlands (7, 20). The average HL score in our study is lower than in any other study. However, any comparison should be made with caution, as the results of the Asian and European studies include on the one hand both male and female and on the other hand only literates.

### Determinants of Women's Level of Health Literacy

**Figure 3:**
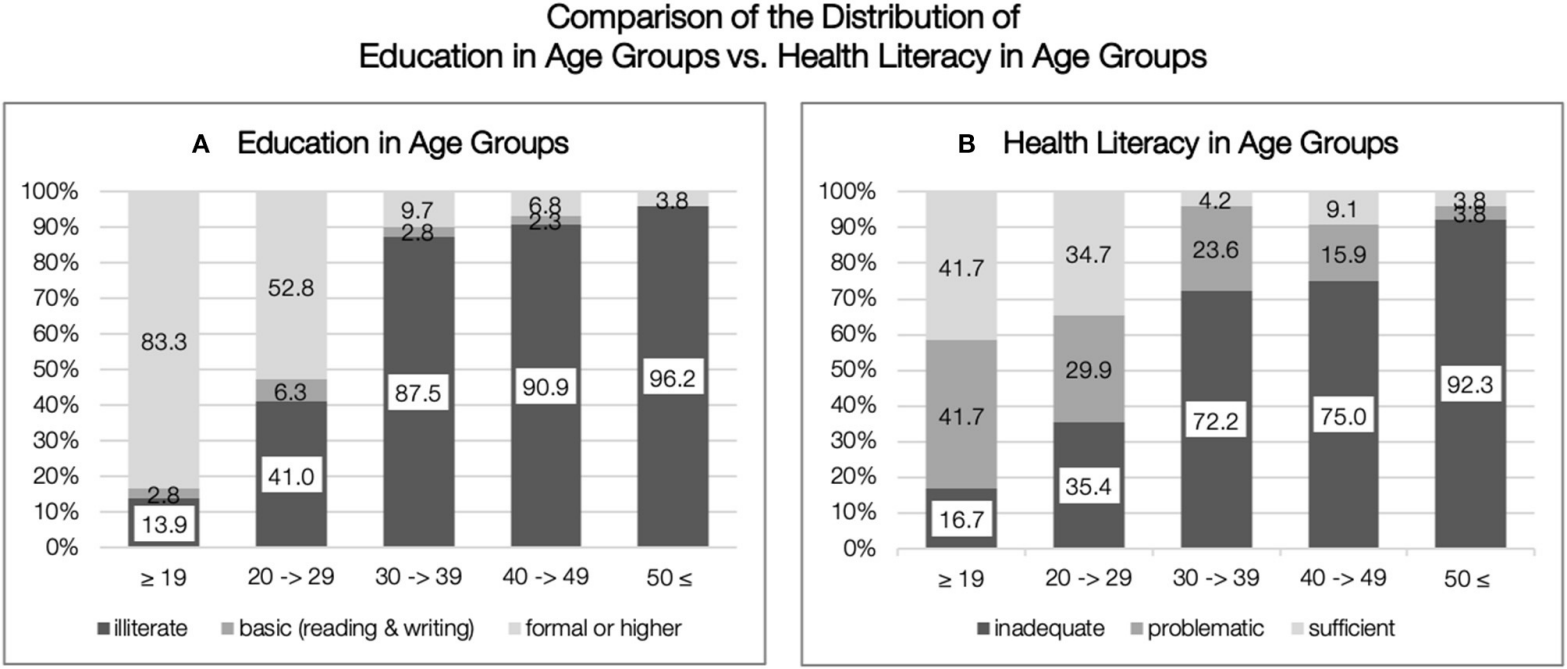
Comparison of the distribution of education in age groups vs. health literacy in age groups. **(A)** Education in age groups. **(B)** Health literacy in age groups.

When analyzing the association of HL with various factors, we found a very heterogeneous picture. Based on raw correlations, HL was associated with age and education. The highest bivariate correlation existed between health literacy and education (*r* = 0.779, *p* < 0.001), followed by age (*r* = −0.462, *p* < 0.001), marital status (*r* = −0.385, *p* < 0.001), not working at home (*r* = 0.378, *p* < 0.001). Women with some education were 73.5 times more likely to have sufficient HL (Odds Ratio (OR) = 73.5 [95% Confidence Interval (*CI*): 22.33, 241.97)] than illiterates. However, in order to be able to explain the correlation of health literacy with age and education qualitatively, a more detailed examination of the sample in the different age groups and their educational levels is necessary at first. Women in Afghanistan are particularly affected by the historical changes in the country and their impact on the education sector. While under Taliban rule women were denied access to schooling, after 2001 massive investments were made in expanding the education system and girls'education. The unequal distribution in access to education is reflected in our sample. Figure 3A shows the distribution of educational attainment per age group. While the share of women with formal and higher education is highest among those under 20 years of age (83.3%), the share of women between 20 and 29 years of age with formal education is still 52.8%, decreasing significantly in the higher age groups. Given this knowledge, we can analyze the distribution of health literacy in the age groups in a more differentiated way (Figure 3B). Again, a noticeable increase in the proportion of women with inadequate health literacy is evident in the age groups of 30. Remarkably, despite the high proportion of young women with formal and higher schooling, not all have equally adequate health literacy. The comparison of the distribution of schooling and health literacy in the age groups invites us to take a more differentiated look at the sample and to explore possible explanations for the large differences not only in the individuals themselves but also in historical changes.

We performed a multivariate linear regression analysis, using education, age, and socioeconomical status (SES) (with marital status, and profession (not working at home) as proxies) as predictors of health literacy. These variables explained up to 60.5% of the variance (adjusted *R*^2^) [χ^2^ (4) = 124.97, *p* = 0.001], as presented in Table 3. Education proved to be the strongest predictor in this regression model and when controlled for education, all other factors were no longer significant.

**Table 3:** Determinants of Women's Health Literacy—multivariate linear regression model.

		** Coefficients**^****a****^				
		**Unstandardized coefficients**		**Standardized coefficients**		
**Model**		**B**	**Std. Error**	**Beta**	***t***	**Sig**.
1	(Constant)	56.678	2.459		23.048	<0.001
	Age	−0.712	0.076	−0.462	−9.314	<0.001
2	(Constant)	28.316	2.383		11.883	<0.001
	Age	−0.108	0.064	−0.070	−1.678	0.094
	Illiterates vs. Educated	24.914	1.416	0.735	17.593	<0.001
3	(Constant)	28.025	2.377		11.788	<0.001
	Age	−0.100	0.064	−0.065	−1.562	0.119
	Illiterates vs. Educated	23.981	1.490	0.708	16.094	<0.001
	Occupation: work at home vs. work outside	3.992	2.061	0.075	1.937	0.054
4	(Constant)	28.341	2.722		10.411	<0.001
	Age	−0.096	0.066	−0.063	−1.454	0.147
	Illiterates vs. Educated	23.920	1.514	0.706	15.795	<0.001
	Occupation: work at home vs. work outside	3.796	2.220	0.071	1.710	0.088
	Marital Status	−0.465	1.946	−0.010	−0.239	0.811

a *Dependent Variable: Health Literacy (measured with the adapted HLS–EU-Q16 on a 4-point-Likert scale)*.

We examined the assumption that people who had easier access to *infrastructure* (transportation and technology) were more likely to be exposed to health-related information and therefore more likely to have higher HL. This assumption is partially supported by our data, when controlled for education and age, only small correlations existed between the level of HL and access to electricity (*r* = 0.152), Internet (*r* = 0.120, *p* = 0.033), and TV (*r* = 0.231, *p* < 0.001). Certain *social/household characteristics* were associated with the HL level, for example, a small positive correlation existed between the number of literate people in the household and HL level (*r* = 0.147, *p* = 0.009). This finding suggests the extraordinary importance of education not only for the individual but also at the family or household level. Furthermore, HL was positively associated with occupation, as women who worked at home were more likely to have lower HL level than women who worked elsewhere (e.g., teacher, NGO employees, and others) [χ^2^ (6) = 68.399, *p* < 0.001, Φ = 0.461].

### Health Literacy and Help-Seeking Behavior and Health-Related Behavior

We explored the relationship between HL and other factors that could be influenced by HL (e.g., help-seeking and health-related behavior) (see Table 4).

**Table 4:** Relationship of health literacy level and health practices.

	**Total**	**Inadequate HL (*N* = 166; 51.6%)**	**Problematic HL (*N* = 83; 25.8%)**	**Sufficient HL (*N* = 73; 22.7%)**	**Significance^**(a)**^**
**HELP SEEKING BEHAVIOR**					
**Where to go in case of sickness** (%) (*N* = 322)_doctor/health center (D)_traditional treatment (T)_mullah (M)_local expert (LE)	D: 74.2 T: 21.7 M: 3.4 LE: 0.6	D: 69.3 T: 24.1 M: 6.0 LE: 0.6	D: 81.9 T: 16.9 M: 1.2 LE: 0.0	D: 76.7 T: 21.9 M: 0.0 LE: 1.4	ns
**Where to take an unconscious woman when she is pregnant** (%)					
(*N* = 322)_doctor/health center (D)_local nurse (LN)_mullah (M)	D: 87.6 LN: 4.7 M: 7.8	D: 81.3 LN: 4.8 M: 13.9	D: 94.0 LN: 3.6 M: 2.4	D: 94.5 LN: 5.5 M: 0.0	χ^2^ (4) =18.506, *p < * 0.001
**REPRODUCTIVE HEALTH—PREVENTION**					
**Having info on how to prevent unplanned pregnancy** (“Yes”) (%) (*N* = 268)	34.7 (*CI:* 28.97–40.44)	28.3	33.3	60.5	*rpb* =0.128, *p* = 0.037
**Using contraceptives** (“Yes”) (%) (*N* = 268)	29.1 (*CI:* 23.63–34.58)	25.2	25.8	48.8	ns
**Use of contraceptives perceived as a sin** (“Yes”) (%) (*N* = 322)	45.03 (*CI:* 39.57–50.49)	62.7	31.3	20.5	*rpb* = −0.366, *p < * 0.001
**Being aware of complexity of pregnancy** (“Yes”) (%) (*N* = 268)	19 (*CI:* 15–24)	10.1	24.2	46.5	*rpb* = 0.303, *p* < 0.001
**PREGNANCY PERIOD**					
**Number of pregnancies** (Nr.) (*N* = 322)	3.45 (*R*: 0–16)	4.92 (*R*: 0–16)	2.47 (*R*: 0–11)	1.21 (*R*: 0–6)	*r* = −0.474, *p* < 0.001
**Doctor visits during pregnancy** (“Yes”) (%) (*N* = 268)	51.5 (*CI:* 45–58)	37.7	63.6	83.7	*rpb* = 0.296, *p* < 0.001
**Number of Doctor visits** (Nr.) (*N* = 246)	2.91 (*R*: 0–15)	1.99 (*R*: 0–9)	4.00 (*R*: 0–15)	4.82 (*R*: 0–9)	*r* = 0.380, *p* < 0.001
**Child born in health center** (‘Yes') (%) (*N* = 246)	51.2 (*CI:* 45–58)	38.3	60.3	87.2	*rpb* = 0.375, *p* < 0.001
**Number of children born in health center** (Nr.) (*N* = 246)	1.2 (*R*: 0–8)	1.06 (*R*: 0–8)	1.36 (*R*: 0–5)	1.49 (*R*: 0–5)	*r* = 0.210, *p* < 0.001
**FEEDING THE CHILD**					
**Importance of breastfeeding**					
(very/important) (%) (*N* = 322)	91.4	92.8	98.8	98.6	*r* = 0.184, *p* < 0.001
**First breastfeeding after birth** in					
hours (*N* = 267)	17.36 (*R:* 0–73)	22.52 (*R:* 0–73)	11.3 (*R:* 0–73)	7.86 (*R:* 0–73)	*r* = −0.358, *p* < 0.001
**Breastfeeding is the first nutrition**					
**given to a newborn** (%) (*N* = 268)	70.5	63.5	74.2	90.7	*r* =0.167, *p* < 0.001
**NUTRITION**					
**Vegetable consumption** (%) (*N* = 322)					
_daily (D)_weekly (W)_monthly (M)_seasonally (S)_not consuming (N)	D: 18.3 W: 9.0 M: 8.4 S: 50.0 N: 14.3	D: 12.0 W: 5.4 M: 7.2 S: 55.4 N: 19.9	D: 22.9 W: 12.0 M: 4.8 S: 53.0 N: 7.2	D: 27.4 W: 13.7 M: 15.1 S: 34.2 N: 9.6	χ^2^ (8) = 30.617, *p* < 0.001
**Fruit consumption** (%) (*N* = 322)					
_daily (D)_weekly (W)_seasonally (S)_not consuming (N)	D: 14.1 W: 10.1 S: 63.6 N: 12.1	D: 7.0 W: 6.3 S: 69.6 N: 17.1	D: 15.8 W: 9.2 S: 69.7 N: 5.3	D: 30.2 W: 20.6 S: 41.3 N: 7.9	χ^2^ (6) = 39.90, *p* < 0.001

CI, Confidence Interval; M, Mean; N, absolute Numbers of respondents; Nr, Number stated by the respondents; ns, not significant; R, range; r, correlation coefficient; rpb, point–biserial correlation coefficient.

(a) *Statistical significance was calculated in three ways. For variables with multiple responses, the chi–square was calculated. For variables with “Yes" vs. no or numerical responses, the variable was correlated with the interval–scaled sum score of HL*.

Regarding *help-seeking behavior*, we asked the participants what they would do in certain cases. In case of sickness, three out of four (74.2%) women would seek advice from a doctor or a health center. Traditional treatment was also important (21.7%). Additionally, 3.4% of women would consult mullahs (religious persons who take care of mosques and teach Islamic subjects) on health matters, and 0.6% of women local experts (such as elderly, wise, village leaders). The woman's choice for these experts was not associated with the HL level. In case a pregnant woman is unconscious, almost 9 out of 10 (87.6%) women recommended taking the woman to the doctor or health center; 7.8% would recommend the mullah and 4.7% the local nurse. The recommended person is associated with the level of health literacy [χ^2^ (4) = 18.506, *p* = 0.001]. So, women with sufficient HL go to the doctor more often than expected and women with inadequate HL go to the mullah.

Concerning reproductive health, information on the prevention of unplanned pregnancy (34.75%) was not common among women, but a positive association existed with having information and a higher HL level [*OR* = 3.61 (95% *CI*: 1.84, 7.08)]. Only 29.1% of women reported using contraceptives, while women with sufficient health literacy were 2.81 times more likely (95% Cl: 1.44, 5.49) to use contraceptives than women with problematic and inadequate HL. The use of contraceptives was to a great extent perceived as a sin, with nearly one in two women (45.03%) agreeing with the statement. We found a moderate correlation between evaluating contraceptives as a sin and a lower HL level (*rpb* = −0.366, *p* < 0.001). As a result, women with higher education were 0.24 times more likely to consider the use of contraceptives as a sin (*OR* = 0.24 (95% *CI*:0.18, 0.44)] than illiterate women. Concerning the period of pregnancy and birth, only 19% of women reported to be aware of the complexity of the pregnancy period. The HL level has a moderate positive association with an awareness of the complexity (*rpb* = 0.303, *p* < 0.01). Controlled for education, none of these four items are significant anymore. Table 4 below shows the distribution of family health-related questions overall and in relation to health literacy.

The level of health literacy was associated with the help-seeking behavior during pregnancy. Women with a higher HL level were more likely to seek help from the doctor (*r* = 0.311, *p* < 0.001), to visit the doctor more often (*rpb* = 0.351, *p* < 0.001) and to give birth in a health center (*r* = 0.375, *p* < 0.001) than women with low HL level. The HL level was also significantly associated with breastfeeding behavior. Women with a higher HL level rated breastfeeding as more important (*r* = 0.186, *p* = 0.001), started breastfeeding earlier after the baby's birth (on average after 7.86 h (sufficient HL) compared to 22.52 h (inadequate HL)) and were more likely to breastfeed the child rather giving the child oil or other food [*OR* = 4.88, (95% *CI*: 1.68, 14.15)] than women with a lower HL level.

Eating behavior was also associated with health literacy: women with “sufficient HL” were more likely to drink warm tea (compared to hot or cold tea) and to eat vegetables and fruits on a daily basis. In contrast, women with lower HL were more likely never to eat vegetables or fruit. Controlled for age and education, the association with fruit consumption remained significant, but not with tea or vegetable consumption.

## Discussion

Every research project in Afghanistan faces various difficulties, which are even more challenging in remote areas outside the large cities (e.g., safety, security, corruption, access to regions, illiteracy, unfamiliarity with research, capacity of research assistants, travel restrictions). In light of these challenges, we endeavored to achieve the highest standard of research in the given context while collecting data relevant to research and practice concurrently. Nonetheless, our study is limited in terms of method, context and data collection process, as well as policy relevance. The first limitations are related to the method and instrument used. Consistent with a standardized health literacy assessment, we used the HLS-EU-Q16 questionnaire and assessed the level of health literacy among women at the hospital in Central Afghanistan. However, this questionnaire only captures one side of health literacy the individual's perspective on his/her own skills and abilities and does not assess the demands and complexity of the health system and situation. Therefore, we can only conclude that women's health literacy level is low, but we cannot specify why. Possible explanations for the low level of HL are lack of education, lack of sufficient health centers in this region, high demands of the hospital, lack of health knowledge and health awareness campaigns etc. but this is not empirically proven in longitudinal studies or studies that assess both sides of health literacy (6). Hence further studies are necessary that explore these aspects in detail, describe their relationship and the development over time. Nevertheless, combining a globally questionnaire with locally relevant questions helps to discuss the adequacy of this common health literacy assessment for populations in distinct regions. Although we identified a need to improve health literacy, our data clearly show that health literacy is associated with education and better health practices.

Secondly, limitations are linked to the context, data collection process and sample. As we conducted the interviews orally by hospital representatives in the Shuhada Hospital, we could not completely eliminate reporting bias and social acceptability bias. Therefore, it is likely that women reported more positively, and the actual level of women's health literacy is worse. Due to the fact that we could not carry out a rigorous random sampling across Afghanistan or the province, our sample and the findings are not representative of all of Afghanistan. However, by randomly selecting women in one hospital in Central Afghanistan over an extended period of time, we sought to collect data from women whose characteristics are representative of this area. Comparing our sample characteristics with those of other study populations in the study area and in Afghanistan, we found them to be very similar (42, 43). Thus, our sample is a good representative of women in this remote area and that our data is the best available and generalizable for Ghazni province or even the Hazarajat.

Thirdly, limitations exit with respect to policy relevance. We originally intended to collect representative data on health literacy in Afghanistan to inform the Afghan government about the level of health literacy among the Afghan population, identify vulnerable groups, and support policy making. However, this was not feasible, primarily for security reasons. So, we refrained from conducting a general assessment of health literacy across the country and focused on examining health literacy and associated determinants, as well as health practices in more detail in one region. As the population in the remote region in central Afghanistan is very vulnerable, our data will help on the one hand the government formulate tailored policy recommendations for this highly at-risk group and on the other hand assist health professionals in this area to address the specific needs identified. Furthermore, this targeted approach is consistent with our research ethic that research should not be conducted merely for the sake of research, but that it should also directly contribute to making a difference in the lives of the study participants.

In light of these challenges, the consequential decisions and resulting limitations, our study was the most feasible and offers unique insights into health literacy, health practices, quality of life, and religious beliefs of women in Central Afghanistan. To provide a detailed analysis of health literacy and related factors, we choose to focus on health literacy in this article and discuss quality of life and spiritual and religious beliefs in other articles (35, 44).

Our study shows that the HL level and literacy rates of women in Afghanistan are low and among the lowest rates compared to other countries worldwide. To the best of our knowledge, only one other study has examined the health literacy of Afghans. Wångdahl et al. interviewed refugees shortly after their arrival in Sweden, including 33 participants from Afghanistan. Of these (male!) Afghans, 29.9% had inadequate, 40.7% problematic and 29.6% sufficient health literacy as measured by the HLS-EU-16 questionnaire (45). However, our female sample differs substantially from the participants in the Swedish study, thus limiting comparison. Generally, our female sample in Afghanistan has lower levels of HL than other countries which is in line with the empirical evidence that lower education levels are associated with lower HL. This was also observed among the male heads of household in Central Afghanistan (35). Nevertheless, it remains surprising that although the mean level of health literacy is low, it is comparably higher than expected. The comparison between European and Asian countries reveals that the included Asian countries have, on average, slightly lower levels of health literacy than European countries measured with the HLS-EU-Q16 developed in and for Europe. Additionally, the studies show that also European countries e.g., Bulgaria, report lower HL than other Asian countries, so a mere comparison of continents is not sufficient, but it points out that a more profound analysis is helpful. A comparison between the Asian countries and Afghanistan also shows that the countries included in the Asia HL survey are neither war-torn, nor among the least developed countries, nor do they have a large number of illiterate people like Afghanistan. This illustrates that the study populations and the contexts in each country differ already significantly from the other countries, making a true comparison nearly impossible. Given the lack of research on health literacy in least developed countries, we could only compare our findings with the data available in the European and Asian surveys and now empirically confirm the assumption that women in Afghanistan have very low levels of health literacy. Yet, to understand this low number properly, we need to include more contextual factors and interpret this quantitative data qualitatively. A first explanation for the low level of health literacy is that educational attainment, an important determinant of health literacy, is generally low among women in Afghanistan. In addition, because health centers are sparse, access to health information is low and skills to engage with health (system) related information are rarely systematically developed. Further studies are needed.

In general, our findings are consistent with the extensive scientific evidence, indicating that determinants of health, particularly educational attainment, are major factors explaining the level of HL (7). Contrary to other studies (4, 20), our study revealed only a minor correlation between HL and age. The relationship between education, age and health literacy may be part of a cohort effect, as younger women had more access to the Afghan education system than older women (29, 46). Moreover, social status is significantly correlated with HL, which is evident from the correlation between structural resources and self-reported health literacy. This confirms the relationship between social inequalities and health inequalities (47, 48).

Additionally, this study provides unique insights into everyday health topics such as reproductive health, help-seeking behavior and dietary pattern of women in Ghazni Province. These findings may help explain the high rates of under-five mortality (91.1/1,000), maternal mortality (396/100,000), and stunted children (40.9%) (49). The study could also identify various relationships between health-related behavior and self-reported health literacy, by considering structural factors.

While illiterate people are usually excluded in research studies due to existing barriers, we have succeeded in including illiterates by means of face-to-face interviews. In accordance with the traditional understandings of functional HL and its measures such as TOHFLA or REALM, illiterates should have virtually no health literacy (50). Though a substantial number of illiterates in our study reported problematic HL, a considerable number of them also reported sufficient HL. This is plausible as the HLS-EU-Q measures comprehensive HL that goes beyond functional HL and focuses on tasks and interaction between people and social services (6, 51). Furthermore, the health systems in low-income countries may be absent or less accessible, located far from many people, often very simple and thus easier to navigate than in high-income countries whose health system might provide more services, easier accessible and better specialized. In our study, strong associations exist between the availability of a nearby health center and the likelihood of seeking medical care from a health professional. For example, having a hospital makes people more likely to seek care there and less likely to seek help from a mullah or to use traditional treatment. Some people, despite having almost no formal education, have sufficient competencies to behave healthy in everyday life. In this sense, the study supports the argument for universal precaution, which takes into account that all people should be treated as at risk for low HL unless they show sufficient HL (21). The response pattern to survey items such as “understanding easily what your friends say” reveals the personal (verbal) interaction with a relevant person, which can strongly influence health literacy and which, especially in developing countries, formal education is unlikely to have the same importance for everyday life practice as in industrialized countries. Therefore, health literacy should be better understood as a social practice and a shared skill (52).

Many health policy approaches assume that improving access to technical infrastructure in remote areas leads to an improvement in health literacy. This assumption could not be confirmed in our data because, first, access to information and communication technology in rural and remote areas of Afghanistan is low generally (42) and, second, we found no link between access to it and a woman's self-reported HL (if controlled for education and age). Hence, based on the results of this study, this assumption is not complex or differentiated enough. According to studies from highly industrialized countries, Internet access does not automatically determine the level of HL (or e-health literacy), as it depends on the individual's media use and on tools for adequately assessing (e) health literacy.

Lastly, this research also provides new insights into the development of General Generalized Resistance Resources (GRR) to cope with adversity and to combat chaos (entropy) (53). Health literacy, a macrosocial GRR, can be structured according to contexts other than traditional learning and curative environments. As noted earlier, a considerable number of illiterates displayed sufficient HL. Despite adversities, these women were able to build their sense of coherence (54) (referred to as a critical asset to fight chaos) based on daily experiences in which they could comprehend, manage, and invest in their progress toward the ease-pole of the dysfunctionality-functionality continuum (53). Not surprisingly, the network and verbal interaction increase these women's GRR. This will determine either a decline or a relative increase in health experiences toward the maximum ease in an environment such as Ghazni, will be a learning lesson in overcoming adversity (55).

A salutogenic perspective will shed new light on the interplay between life orientation and the development process of health literacy, particularly in countries of the Global South.

## Conclusion

The study aimed to increase evidence for HL in Asia and crisis-affected countries, by providing novel data on health literacy in Afghanistan. Compared to other Asian countries, the self-reported HL level of women in the Ghazni Province in Afghanistan is low. This could be explained by the high illiteracy rate, a consequence of political events in Afghanistan. Although (formal) level of education is the strongest predictor of a person's individual HL level, this study clearly reveals that illiterate people can be health literate and behave healthily.

This cross-sectional study illuminates contextual factors, various health-related behaviors, and health literacy and their interrelationship. The forthcoming studies and interventions contribute to enhancing our understanding of the complex relationship between literacy, HL, health behavior, quality of life etc. However, cross-sectional studies such as this cannot inform us about the process of acquiring and shaping health literacy or health behavior. In our view, more research—including ethnographic research—is needed both to thoroughly investigate the relationship between contextual factors, health literacy, help-seeking behavior, and health-related practices in everyday life and to explore the development of health literacy and behavior in daily lives. Hereby, a salutogenic understanding of people's competencies and resources of action is promising. Based on this deeper understanding, further interventions to improve health literacy in schools and in daily practice should be developed. The recommendation to implement more interventions to further improve women's HL is in line with the Afghan Government's strategy: “gender mainstreamed in all health promotion interventions and effective health literacy messaging to women and girls” (33). Finally, access to infrastructure and electronic devices is not automatically linked to higher levels of HL, hence improving new technologies in Afghanistan cannot be the stand-alone strategy for improving health literacy. A more comprehensive strategy is needed encompassing health literacy as a shared social practice and as a complex and urgent public health challenge for the people of Afghanistan that should be addressed.

## Data Availability Statement

The raw data supporting the conclusions of this article will be made available by the authors, without undue reservation.

## Ethics Statement

Ethical review and approval was not required for the study on human participants in accordance with the local legislation and institutional requirements. Written informed consent from the participants' legal guardian/next of kin was not required to participate in this study in accordance with the national legislation and the institutional requirements.

## Author Contributions

SH: data analysis and writing the article. AJ: data collection and reviewing the article. EJ: data collection. LS-N: reviewing the article. KS: reviewing the article. DS: reviewing the article. UB: writing the article. All authors contributed to the article and approved the submitted version.

## Conflict of Interest

The authors declare that the research was conducted in the absence of any commercial or financial relationships that could be construed as a potential conflict of interest.
